# Enhancing DNA Vaccine Delivery Through Stearyl-Modified Cell-Penetrating Peptides: Improved Antigen Expression and Immune Response In Vitro and In Vivo

**DOI:** 10.3390/vaccines13010094

**Published:** 2025-01-20

**Authors:** Sheng Jiang, Cheng Zu, Bin Wang, Yiwei Zhong

**Affiliations:** 1 Shanghai Institute of Infectious Disease and Biosecurity, Fudan University, Shanghai 200032, China; jimjiang09@163.com (S.J.); 21111010095@m.fudan.edu.cn (C.Z.); 2 Key Laboratory of Medical Molecular Virology (MOE/NHC/CAMS), School of Basic Medical Sciences, Fudan University, Shanghai 200032, China

**Keywords:** DNA vaccine, cell-penetrating peptide, in vitro expression, in vivo expression, antibody response, T cell response

## Abstract

Background: Inefficient cellular uptake is a significant limitation to the efficacy of DNA vaccines. In this study, we introduce S-Cr9T, a stearyl-modified cell-penetrating peptide (CPP) designed to enhance DNA vaccine delivery by forming stable complexes with plasmid DNA, thereby protecting it from degradation and promoting efficient intracellular uptake. Methods and Results: In vitro studies showed that S-Cr9T significantly improved plasmid stability and transfection efficiency, with optimal performance at an N/P ratio of 0.25. High-content imaging revealed that the S-Cr9T–plasmid complex stably adhered to the cell membrane, leading to enhanced plasmid uptake and transfection. In vivo, S-Cr9T significantly increased antigen expression and triggered a robust immune response, including a threefold increase in IFN-γ secretion and several hundred-fold increases in antibody levels compared to control groups. Conclusions: These findings underscore the potential of S-Cr9T to enhance DNA vaccine efficacy, offering a promising platform for advanced gene therapy and vaccination strategies.

## 1. Introduction

DNA vaccines have emerged as a promising innovation in immunology, offering a flexible platform to combat a wide range of infectious diseases. Unlike conventional vaccines that use live-attenuated or inactivated pathogens, DNA vaccines introduce a segment of genetic material—typically a plasmid—that encodes key antigens from the target pathogen [1,2,3,4,5]. Once inside the host, the plasmid directs cellular machinery to synthesize these antigens, triggering an immune response [6,7,8]. This capability to quickly adapt to new pathogens makes DNA vaccines especially valuable in responding to emerging infectious diseases, such as rapidly mutating viruses [9,10].

Despite this potential, the clinical application of DNA vaccines has been limited by their relatively low immunogenicity. A major challenge lies in their poor cellular uptake and expression of encoded antigens [11]. Several delivery strategies, such as liposomes and electroporation, have been developed to enhance DNA uptake. However, while these methods improve DNA delivery, their impact on boosting antibody production and T cell immunity is often modest, and the inconvenience associated with electroporation limits its broad application [12,13,14,15].

Cell-penetrating peptides (CPPs) have garnered attention as an alternative delivery method due to their unique ability to facilitate the intracellular delivery of biomolecules, including nucleic acids [16,17]. Composed of short amino acid sequences, CPPs can cross cellular membranes, promoting the cellular uptake of therapeutic agents such as DNA plasmids [18,19,20]. Among them, the Tat peptide, derived from the HIV-1 transactivator of transcription protein, is a well-known CPP rich in arginine residues that efficiently penetrates cell membranes and delivers various cargos [21,22,23,24]. However, challenges remain in optimizing CPPs for plasmid delivery. Many CPPs suffer from enzymatic degradation and lack the structural integrity necessary for stable complex formation with plasmids [25,26]. While CPPs have been widely explored for the delivery of nucleic acids, such as DNA or RNA, their application specifically in delivering plasmid DNA (pDNA) remains improved and underexplored. The unique properties of plasmid DNA, such as its larger size and structural complexity, make its delivery more challenging compared to smaller nucleic acids. Advanced CPP modifications, including branched or hydrophobic modifications, aim to overcome these limitations by improving both stability and delivery efficiency. Advanced CPP modifications, such as hydrophobic moieties or disulfide bonding, may provide the necessary stability and delivery efficiency to overcome these challenges [27,28,29,30].

In this study, we investigate the design and application of a novel stearyl-modified CPP, S-Cr9T, for the delivery of DNA vaccines. By integrating hydrophobic moieties and cysteine-mediated disulfide bonding, we aimed to improve the stability of the CPP–plasmid complex and enhance both in vitro and in vivo delivery efficiency. Our results demonstrate the ability of S-Cr9T to form stable complexes with plasmid DNA, significantly improving cellular uptake, transfection efficiency, and immune response.

## 2. Materials and Methods

### 2.1. Cell Transfection

The HEK293T cell (provided by Professor Lu Lu, Fudan University) density reached 90%, and the culture supernatant was replaced with serum-free MEM medium 30 min before transfection. The transfection reagent (TransIT^®^-LT1, Mirus, Madison, WI, USA) and the plasmid were mixed in the MEM medium at a ratio of 2:1, allowed to stand for 5 min, then gently mixed and allowed to stand for another 10 min before adding dropwise into the target well of the cell culture plate. The culture was incubated at 37 °C with 5% CO_2_ for 6 h. The supernatant was then discarded and a complete medium (DMEM with 10% FBS) was added. Cells were harvested 24, 48, and 72 h post transfection to determine protein and mRNA expression levels by repeated freeze–thaw cycles. The reagents used in cell culture, including RPMI1640 medium, DMEM medium and Tris-HCl buffer (TBS), phosphate-buffered saline (PBS), and Hepes buffer, were all from Meilunbio, Dalian, China; fetal bovine serum (FBS), trypsin-EDTA, penicillin-streptomycin-amphotericin B solution, and mycoplasma antibiotics BIOMYC were all purchased from Biological Industries, Kibbutz, Israel.

### 2.2. Preparation of Peptide Gel

Under sterile conditions, the molarity of the CCPs was calculated based on their molecular weights and they were dissolved in 1 mL of 98% DMSO at room temperature to make a 100 mM stock solution, which was stored at −20 °C. A 30 µL aliquot of the 100 mM peptide stock solution was added to 70 µL of PBS to obtain a 30 mM solution, which was then incubated on a shaking platform at 200 rpm at room temperature for 6–12 h. After observing the formation of a solid gel, different volumes of 5 mM Hepes buffer at pH = 7.4 were added to terminate the reaction based on the corresponding CPPs (S-Cr9T: 220 μL, S-Btat: 237 μL, Cr9T: 213 μL, and R9Tat: 447 μL), resulting in a final peptide concentration of 5 mg/mL. The gel was then stored at 4 °C. In this study, all synthesized peptides, except the S-Cr9T, which underwent synthesis and stearoyl-C’ modification by Ontores (Hangzhou, China), were synthesized by GenScript (Nanjing, China) with a purity of ≥90%. The peptides synthesized were Tat (YGRKKRRQRRQ), R9Tat (GRRRRRRRRRPPQ), Btat (CYGRKKRRQRRQCYGRKKRRQRRQC), and Cr9T (CGRRRRRRRRRPPQCGRRRRRRRRRPPQC).

### 2.3. Peptide-Encapsulated Plasmid Transfection

Under sterile conditions, a peptide solution at 5 mg/mL stored at 4 °C was mixed with 10 μg of plasmid pcDNA3.1-EGFP (provided by Advaccine Biopharmaceuticals, Co., Ltd., Suzhou, China) in MEM medium at different N/P ratios (where the N/P ratio refers to the molar ratio of positive charges “N” contributed by the cell-penetrating peptide to the negative charges “P” provided by the plasmid DNA) and incubated at room temperature for 30 min. Subsequently, 100 µL of this mixture was subjected to agarose gel electrophoresis and particle size measurement was performed with the ZetaSizer (Malvern Panalytical, Almelo, The Netherlands). The remaining 100 μL containing 5 μg of plasmid was added dropwise to the culture supernatant of a 24-well plate (90% confluence of HEK293T cells) and incubated at 37 °C with 5% CO_2_ for 6 h. After incubation, the original culture supernatant was replaced with a complete medium (DMEM with 10% FBS), and the expression of fluorescent proteins was observed under a fluorescence microscope (Thermo, Waltham, MA, USA) 48 h after transfection.

### 2.4. Serum Suppression Transfection Assay

Under sterile conditions, CPP solution at 5 mg/mL was mixed with 10 ug of plasmid solution at various N/P ratios. Lipid-based transfection reagent TransTL was mixed with 10 μg of plasmid at a ratio of transfection reagent to plasmid of 2:1 in MEM medium, incubated for 30 min at room temperature. Each 100 µL of the mixture was added dropwise to 900 µL MEM complete medium with or without 10% FBS in a 24-well plate (90% confluence of HEK293T cells). After 6 h of incubation at 37 °C and 5% CO_2_, the original culture supernatant was replaced by the complete medium. The expression of fluorescent proteins was observed under a fluorescence microscope 48 h later.

### 2.5. High-Content Imaging Assay

Until they attained 50% confluence, HEK293T cells were seeded in 24-well plates. Following a careful discarding of the original culture supernatant, 300 µL of Dulbecco’s Phosphate-Buffered Saline (DPBS) was added to completely cover the bottom of the culture dish. The DPBS was disposed of following a minute of standing. Staining the cells for 10 min requires adding 100 µL of Hochest 33342 diluted with DPBS (1 mg/mL). DMEM medium free of serum was added to the staining solution after mixing. First, the plasmids (pNL4.3-luc) were diluted 1:12.5 with PBS and Picogreen (i.e., E. 10 min were spent staining in the dark at room temperature (12.5–2.5 µL of staining solution, or 1 µg of plasmid). Then, at various N (peptide)/P (plasmid) ratios (transfection reagents were mixed with plasmids at a 2:1 ratio), the stained plasmids were combined for incubation with cell-penetrating peptides. The culture supernatant in 24-well glass bottom plates was then mixed with 100 µL of the mixture containing 5 µg of plasmid total, added dropwise. Afterward, the 24-well plate was placed in a high-content imaging system (Operetta CLS High-Content Analysis System, PerkinElmer, Waltham, MA, USA), which was configured to operate at 37 °C and 5% CO_2_. To track the plasmid entry process into the cells, images were captured after hours 0 and 1, 2, 3, and 4. The quantity of plasmids attached to cells and to specific cells was additionally counted by the high-content imaging system.

### 2.6. Plasmid Extraction and Purification

Plasmids were extracted using a large endotoxin-free plasmid extraction kit according to the manufacturer’s instructions (Magen Biotechnology Co., Ltd., Guangzhou, China). Before extraction, 60 mg of RNase was added to the P1 reagent. The bacterial culture was processed by centrifugation and mixed sequentially with reagents P1, P2, and P3 to lyse cells, clear the lysate, and precipitate DNA. The supernatant obtained after centrifugation was treated with an endotoxin removal reagent and then subjected to a series of steps including isopropanol precipitation, adsorption column purification, washing, and elution to concentrate the plasmids. Finally, the plasmid solution was treated with sodium acetate and ethanol, washed with cold ethanol, and resuspended in SSC solution to prepare the stock solution, which was stored at −20 °C.

### 2.7. Mouse Immunization

Eight-week-old female BALB/c mice were immunized intramuscularly (I.M.) three times at two-week intervals, with a plasmid expressing ovalbumin antigen (pVAX-OVA, the plasmid was previously validated in our lab [31]). Serum samples were collected on day 42 for ELISA analysis to assess antibody levels. Additionally, single-cell suspensions of splenocytes were isolated from mice sacrificed on day 42 for ELISPOT analysis to evaluate IFN-γ secretion.

### 2.8. IFN-γ ELISPOT Assay

After the mouse was euthanized, the spleen was removed and homogenized in PBS. The homogenate was centrifuged, and the red blood cells were fast lysed using a lysis buffer. After washing, single cell suspensions of splenocytes were resuspended in complete medium or PBS for further use.

Single cell suspensions of splenocytes were prepared in RPMI1640 complete medium and added to an ELISPOT plate reader (Dakewe Biotech Co., Ltd., Shenzhen, China) pre-coated with an anti-IFN-γ capture antibody. For the experimental group, 1 × 10^6^ splenocytes per well were stimulated with 1 μg/mL OVA_257–264_ peptide. In the positive control group, 1 × 10⁵ splenocytes per well were stimulated with PMA/ionomycin. The plates were incubated for 12 h at 37 °C in a 5% CO₂ incubator. Spots were developed and analyzed using an AID reader (AID, Strassberg, Germany).

### 2.9. Enzyme-Linked Immunosorbent Assay (ELISA)

OVA-specific IgG binding antibodies were detected using a standard ELISA procedure. Briefly, 96-well plates were coated with OVA (Sigma-Aldrich, St. Louis, MO, USA) at a concentration of 1 µg/mL in coating buffer (0.05 M Carbonatebicarbonate buffer, pH 9.6) and incubated overnight at 4 °C. After blocking with 1% BSA in coating buffer for 1 h at room temperature, serum samples were serially diluted and added to the wells. Samples were incubated for 2 h at room temperature. Following extensive washing, the secondary HRP-conjugated anti-mouse IgG antibodies (Abclonal, Woburn, MA, USA) were added and incubated for 1 h at room temperature. The plates were developed using TMB substrate solution, and the reaction was stopped with 2M H_2_SO_4_. Absorbance was measured at 450 nm using a microplate reader (xMark, Bio-Rad, Hercules, CA, USA).

### 2.10. In Vivo Bioluminescence Imaging

Mice were injected intramuscularly (i.m.) with a mixture of CCPs and pNL4.3-luc (the construct was obtained from Professor Lu at Fudan University) at different concentrations and subjected to in situ electroporation. On days 3, 7, and 14 postimmunization, bioluminescence imaging was performed by injecting luciferin, anesthetizing the mice, and recording fluorescence intensity at the injection sites with an in vivo imaging system (IVIS, PerkinElmer, Waltham, MA, USA).

### 2.11. Statistical Analysis

All data analysis was performed using GraphPad Prism 8 software. Data were presented as mean ± standard error. Unpaired Student’s *t*-test was used to analyze the differences between the two groups, while two-way ANOVA and multiple comparison tests were used to analyze the differences between different groups. Significance levels were denoted as * *p* < 0.05, ** *p* < 0.01, *** *p* < 0.001, and **** *p* < 0.0001.

## 3. Results

### 3.1. Design of Cell-Penetrating Peptides and Formation of Cell-Penetrating Peptide–Plasmid Complexes

**Figure 1:**
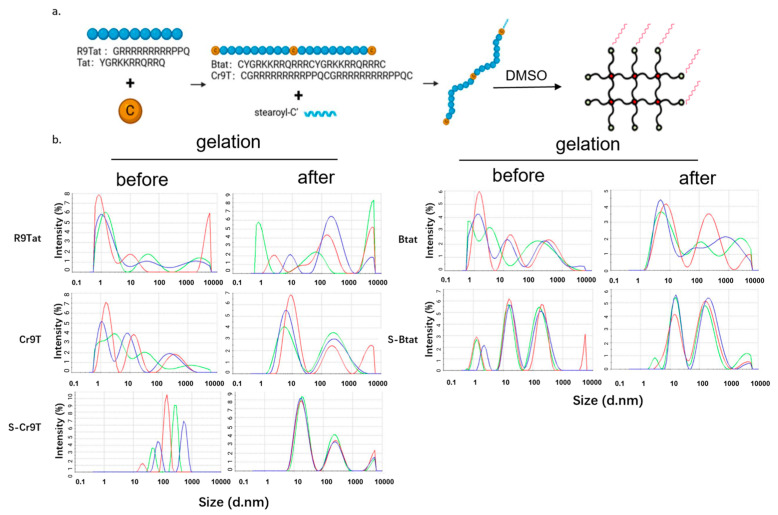
Schematic representation of cell-penetrating peptide (CPP) design and gelation. (**a**). A schematic diagram represents the CPP sequences, design, and gelation process. R9Tat and Tat peptides were modified by introducing cysteine (C), resulting in the formation of Cr9T and Btat. Subsequently, Cr9T was further modified through the attachment of stearoyl-C’, yielding to the final S-Cr9T peptide. The CPPs were composed of Tat, Btat, Cr9T, and S-Cr9T, and their gelations were induced by reacting 30 mM DMSO for 6–12 h dependent on the sequences. (**b**). Dynamic light scattering (DLS) was employed to measure the particle size distribution of the CPP solutions. Prior to gelation, indicated as “before”, each solution was diluted 1000-fold with HEPES buffer. After gelation, indicated as “after”, DLS analysis was repeated to assess any changes in particle size. Different colors in each panel represent independent replicated measurements.

Three CCPs (S-Btat, Cr9T, and S-Cr9T) for DNA vaccines were developed based on previously reported sequences with modifications [31,32]. These included CCPs Tat (YGRKKRRQRRQ), by replacing the R with a Q at the 3′ terminus, and R9Tat (GRRRRRRRRRPPQ), by replacing Ks with Rs from the modified TAT. Cysteines were added to each end and to the middle of TAT and R9T to obtain duplicated forms as listed: Btat (CYGRKKRRQRRQCYGRKKRRQRRQC) and Cr9T (CGRRRRRRRRRPPQCGRRRRRRRRRPPQC). The cysteine residues could form disulfide bonds between each chain and a network structure. To improve coherence on the cell surface, we subsequently added stearyl modifications to the C-terminus of the Btat and Cr9T sequences, resulting in the peptides S-Btat and S-Cr9T. These positively charged polypeptide sequences could interact with negatively charged plasmid DNA to facilitate the formation of stable complexes. Stearyl modification promotes the entry of the peptide–plasmid complex into cells by inserting the stearic acid carbon chain, which helps anchor the complex to the cell membrane (Figure 1a,b, Table 1 and Table 2).

**Figure 2:**
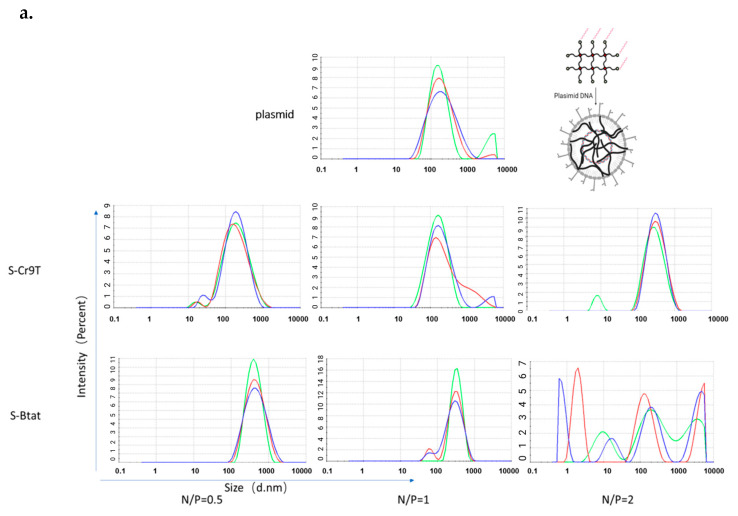

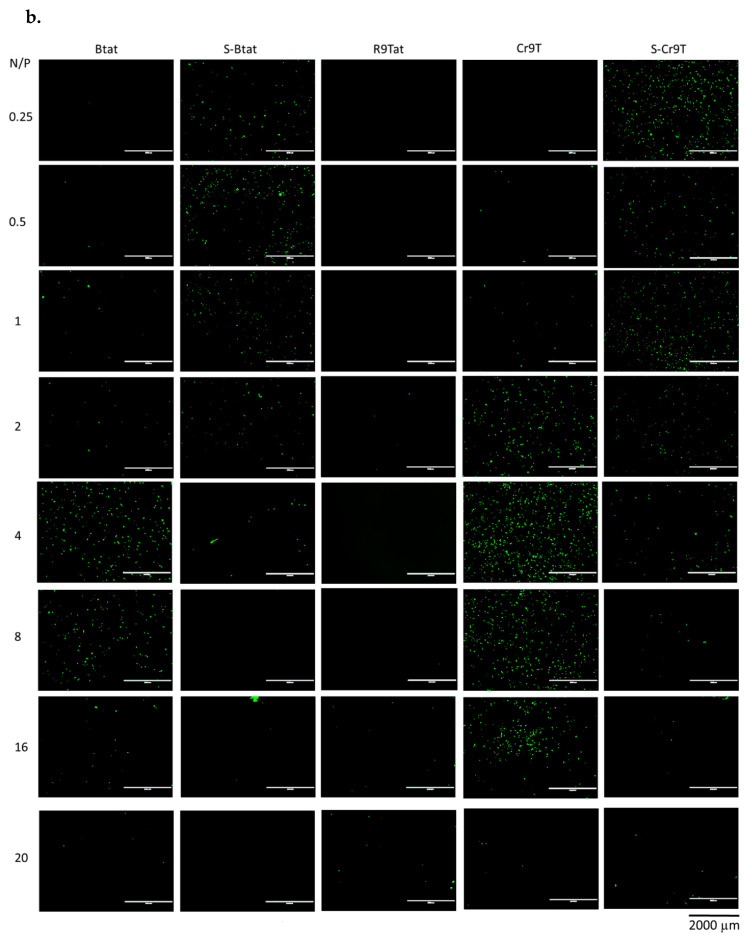
Characterization and transfection efficiency of cell-penetrating peptide–plasmid complexes. (**a**). Ten micrograms of plasmid DNA (pcDNA3.1-eGFP) was mixed with varying amounts of 5 mg/mL of CPPs to achieve specific N/P ratios. The mixtures were incubated for 30 min at room temperature, and 100 μL aliquots were subjected to particle size analysis using a ZetaSizer. The particle size distributions of the S-Cr9T or S-Btat peptide–plasmid complexes at N/P ratios of 0.5, 1, and 2 were determined by dynamic light scattering (Different colors represent independent replicates). (**b**). HEK293T cells were seeded to 90% confluence and subsequently treated with 100 μL of CPP–plasmid complexes for 6 h at 37 °C in a 5% CO_2_ atmosphere. Following a 48 h incubation period in complete medium, the cells were examined for fluorescent expression. The transfection efficiency of various cell-penetrating peptides (CPPs) forming plasmid–CPP complexes at different N/P ratios was assessed. The stearyl modification at the C-terminus of S-Cr9T and S-Btat significantly enhanced transfection efficiency compared to Cr9T and Btat, particularly at low N/P ratios (0.25–2).

After forming gels in vitro, we mixed the diluted gels with plasmids to form cell-penetrating peptide–plasmid complexes. These were then analyzed for their formation and structural stability using DNA-agarose gel electrophoresis and particle size measurement. For S-Cr9t and S-Btat, the N/P ratios were 0.1, 0.2, 0.5, 1, 2, 4, and 8, while the ratios for Cr9t, Btat, and R9Tat were 0.5, 1, 2, 4, 8, 16, and 20. Plasmids were observed to be encapsulated at different N/P ratios. Btat was completely encapsulated at an N/P ratio of 0.5. S-Cr9T and S-Btat began to encapsulate at an N/P ratio greater than two, whereas S-Cr9T was completely encapsulated at an N/P ratio of eight versus Cr9T at an N/P ratio of four. However, R9Tat showed plasmid DNA in the gel well, indicating that the R9Tat–plasmid formed a larger complex with a strong bonding that blocked DNA mobility. This suggests that CCPs (except R9Tat) can effectively encapsulate plasmids and form CCP–plasmid complexes at room temperature (Figure S1b–d). Since reduced glutathione (GSH) can break disulfide bonds, we mixed peptide–plasmid complexes (N/P = 3) with 10 mM reduced GSH solution and incubated for 30 min at room temperature to break the disulfide bonds of the CCPs and release the plasmids. Gel electrophoresis results (Figure S1e,f) showed that plasmids were released from the S-Cr9T, Cr9T, R9Tat, and Btat complexes upon reaction with 10 mM reduced GSH. We also measured the particle sizes of peptide–plasmid complexes (Table 3 and Table 4). However, since only S-Cr9T and S-Btat formed stable structures at different N/P ratios, we only obtained particle size results for these two CCP–plasmid complexes (Figure 2a). S-Cr9T was observed to form CCP–plasmid complexes with plasmids with N/P ratios of 0.5, 1, and 2, and particle sizes in the range of 100–200 nm, and S-Btat formed stable complexes with plasmids with N/P ratios of 0.5 and 1, with particle sizes around 200–300 nm. These results suggest that stearylated CCPs, particularly S-Cr9T, form more stable complexes with plasmids.

### 3.2. CCP–Plasmid Complexes Efficiently Promote Plasmid Transfection In Vitro

The ability of CPP–plasmid complexes to promote efficient plasmid transfection was evaluated in vitro. To assess this, we monitored fluorescence expression following the addition of CPP–fluorescent plasmid complexes to HEK293T cells. After the cells reached 90% confluence, CPP–plasmid complexes (pcDNA3.1-eGFP) were added, and the cells were incubated for 6 h at 37 °C with 5% CO_2_. The medium was then replaced with complete DMEM, and fluorescence was observed 48 h later. We compared the transfection efficiency of various CPP–plasmid complexes by analyzing fluorescence expression (Figure 2b). At N/P ratios starting from 0.25, R9Tat showed no effect on plasmid entry. However, Cr9T, with cysteine modification, demonstrated significantly stronger plasmid transfection capabilities than Btat, particularly at N/P ratios between two and sixteen. Notably, with stearyl modification at the C-terminus, S-Cr9T and S-Btat exhibited superior transfection efficiency compared to Cr9T and Btat, especially at low N/P ratios (0.25–2) (Figure 2b). Maximum efficiency was achieved at an N/P ratio of eight for Cr9T, around four for Btat, and at 0.5 for both S-Cr9T and S-Btat. The short peptides Cr9T and S-Cr9T proved to be significantly more effective at promoting plasmid entry than Btat and S-Btat, whose core peptide is Tat. Based on these findings, Cr9T and S-Cr9T were selected for further studies as the most promising delivery systems for DNA vaccines.

### 3.3. Effect of Serum on Transfection Efficiency

**Figure 3:**
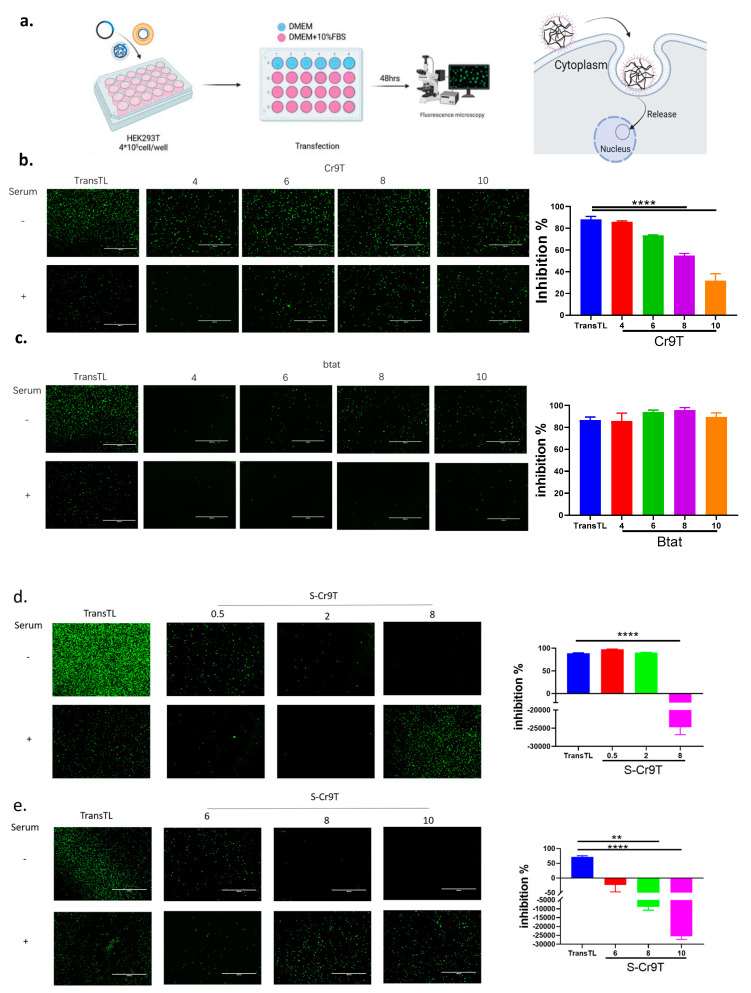
Overcoming serum challenges in DNA transfection with novel CPP–plasmid complexes. (**a**) Serum suppression transfection assay: HEK293T cells were cultured in serum-free DMEM. CPP (5 mg/mL) was mixed with plasmid DNA (10 µg) at various N/P ratios. The lipid-based transfection reagent TransTL (positive control) was added (2:1 ratio) and incubated (MEM, 30 min). Each 100 µL of mixture was added to 900 µL of complete MEM or MEM with 10% FBS (90% confluence). After 6 h (37 °C, 5% CO_2_), the medium was replaced, and fluorescent protein expression was assessed 48 h later to compare efficiency. (**b**,**c**) Fluorescence microscopy images display the expression of fluorescent proteins in HEK293T cells transfected with plasmids complexed with Cr9T or Btat. Notably, at an N/P ratio of 10, Cr9T significantly protected plasmids from serum precipitation compared to Btat (Scale bar: 2000 μm, *n* = 3, **** *p* < 0.0001, unpaired test). (**d**) Lower N/P ratios were tested for the expression efficiency. Fluorescence microscopy images display the expression of fluorescent proteins in HEK293T cells transfected with plasmids complexed with S-Cr9T. (**e**) Higher N/P ratios were tested for expression efficiency. Fluorescence microscopy images display the expression of fluorescent proteins in HEK293T cells transfected with plasmids complexed with S-Cr9T (N/P ratios at 6–10). The ability of S-Cr9T plasmid complexes to support plasmid entry into cells increased significantly (Scale bar: 2000 μm, ** *p* < 0.01, **** *p* < 0.0001, unpaired test).

In vitro transfection studies further demonstrated that CPP–plasmid complexes can successfully enter cells and facilitate plasmid expression. However, it is well established that serum can precipitate DNA and assist the body in eliminating foreign DNA, which presents a significant challenge for effective DNA vaccine delivery. To address this, we conducted transfection experiments by forming CPP–plasmid complexes in DMEM without serum and compared their efficiency to cells cultured in complete medium. The results (Figure 3a) showed that Cr9T was able to protect some plasmids from serum-induced precipitation, facilitating their entry into cells, with the most pronounced effect at an N/P ratio of 10. In contrast, S-Cr9T exhibited high transfection efficiency at low N/P ratios but a diminished ability to protect plasmids from serum precipitation. At higher N/P ratios, S-Cr9T had a reduced capacity to promote plasmid entry but was more effective at preventing serum precipitation. Notably, after the addition of serum, S-Cr9T complexes demonstrated a significant increase in their ability to support plasmid entry into cells (Figure 3b–e).

### 3.4. CCP–Plasmid Complexes Improve Entry of Plasmid into Cells by Anchoring to the Cell Surface

**Figure 4:**
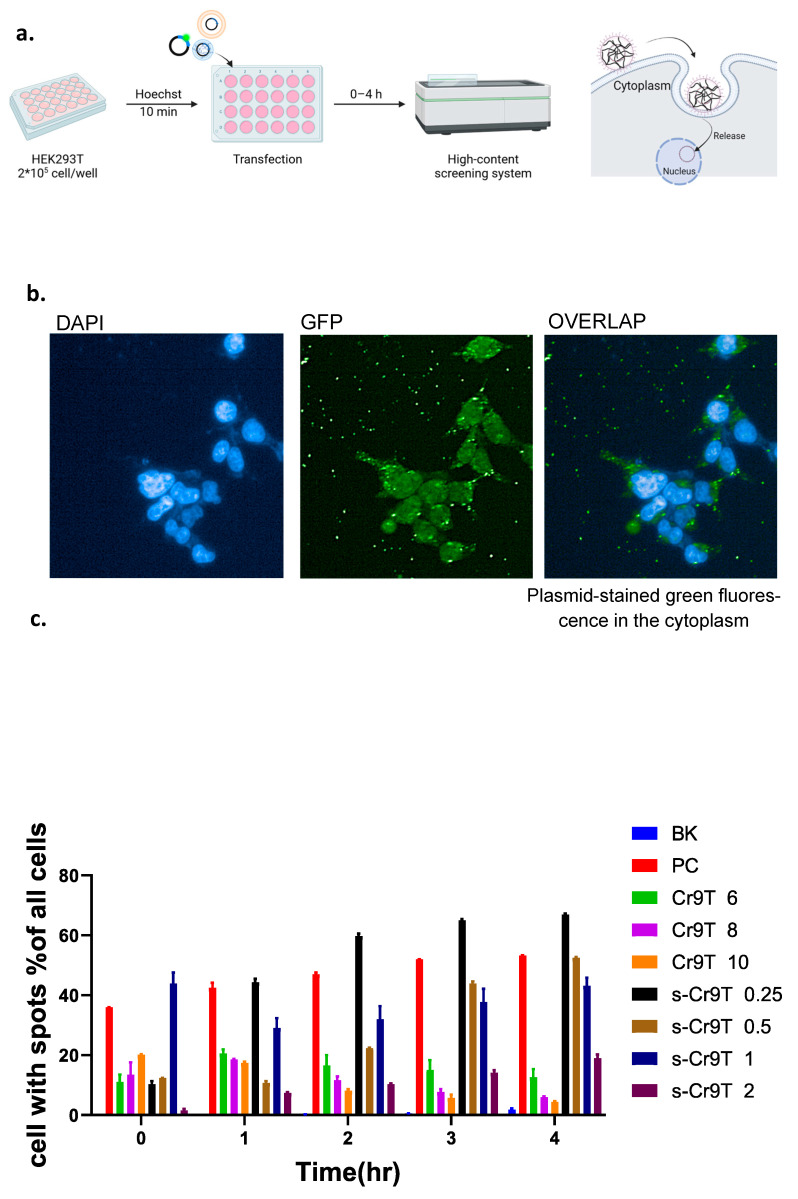

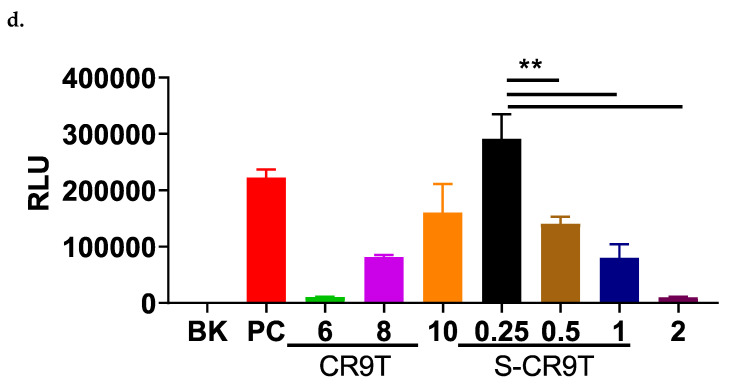
Enhancement of plasmid uptake with stearoyl modified cell-penetrating peptides. (**a**,**b**) Transfection and imaging procedure: Picogreen staining was used to label plasmids, which were then mixed with cell-penetrating peptides (CPPs) to form complexes. Fluorescence images were taken under 37 °C and 5% CO_2_, recording the positions of CPP–plasmid complexes at various N/P ratios and time points (0, 1, 2, 3, and 4 h). DAPI marked nuclei, while the cell membrane was highlighted by bright green fluorescence. Plasmid DNA appeared as green fluorescent spots on the cell membrane. (**c**) Bar charts from high-content fluorescence analysis showing the percentage of cells with plasmid spots for Cr9T–plasmid and S-Cr9T–plasmid complexes. TransIT^®^-LT1 Transfection Reagent served as a positive control. The results indicate the transfection efficiency of the different peptide formulations (BK: blank control, PC: positive control). (**d**). Luciferase activity (pNL4.3-luc expressed), indicating successful transfection results, was derived from the plasmid that expresses luciferase and was previously stained with Picogreen to visualize its entry into cells. Statistical significance was determined using an unpaired test (n = 3, ** *p* < 0.01).

Since CPP–plasmid complexes enhance the entry of plasmids into cells through effective anchoring to the cell surface, we conducted a series of experiments using high-content imaging and dynamic tracking of CPP–plasmid complexes to demonstrate if this is the case. Plasmids were stained with Picogreen and mixed with CPPs to form complexes, which were then added to HEK293T cells. Fluorescent images were captured at 37 °C and 5% CO₂ over a time course (0, 1, 2, 3, and 4 h) to monitor the interaction of the CPP–plasmid complexes with the cells at different N/P ratios (Figure 4a). Using high-content imaging, we visualized the nucleus with DAPI staining and highlighted the cell membrane with bright green fluorescence. Plasmid DNA appeared as distinct green fluorescent spots, enabling the precise quantification of the CPP–plasmid complexes on the cell membrane (Figure 4b). The results showed that Cr9T–plasmid complexes adhered rapidly to the cell membrane, but the number of attached complexes decreased over time, with some remaining visible as bright spots on the membrane (Figure S2a). In contrast, a significant nuclear accumulation of green fluorescence was observed, indicating that Cr9T complexes facilitated the rapid transport of plasmids into the nucleus after a brief membrane interaction. In comparison, S-Cr9T–plasmid complexes behaved differently. Initially, only a small number of S-Cr9T complexes adhered to the cell membrane. However, over time, the number of attached complexes increased significantly, as did the percentage of cells displaying bound complexes. Notably, cells treated with S-Cr9T at an N/P ratio of 0.25 exhibited sustained membrane adhesion even after 4 h, with a higher equilibrium of attached complexes compared to those transfected with the TransIT^®^-LT1 reagent. Upon closer inspection, it was evident that S-Cr9T–plasmid complexes exhibited robust adhesion to the cell surface but did not immediately penetrate the nucleus, likely due to the interaction of the terminal stearoyl groups with the cell membrane. This prolonged adhesion contributed to a delayed nuclear entry compared to Cr9T complexes. Nevertheless, S-Cr9T facilitated more efficient plasmid uptake over a longer time frame, particularly at N/P ratios of 0.25–2, whereas Cr9T complexes were most effective at N/P ratios of 6–10 (Figure 4c and Figure S2b). Following high-content imaging, we examined the transfection efficiency of these complexes. The Cr9T complexes, which exhibited the strongest membrane adhesion at 0 h (N/P = 10), also achieved the highest plasmid transfection efficiency. Meanwhile, S-Cr9T complexes, which showed the highest adhesion at 4 h (N/P = 0.25), delivered the best transfection results at this time point (Figure 4d). These findings suggest a strong correlation between the duration of membrane adhesion and the overall transfection efficiency of the CPP–plasmid complexes. 

### 3.5. Improvement of Intracellular Expression of Antigens by S-Cr9T–Plasmid Complexes

**Figure 5:**
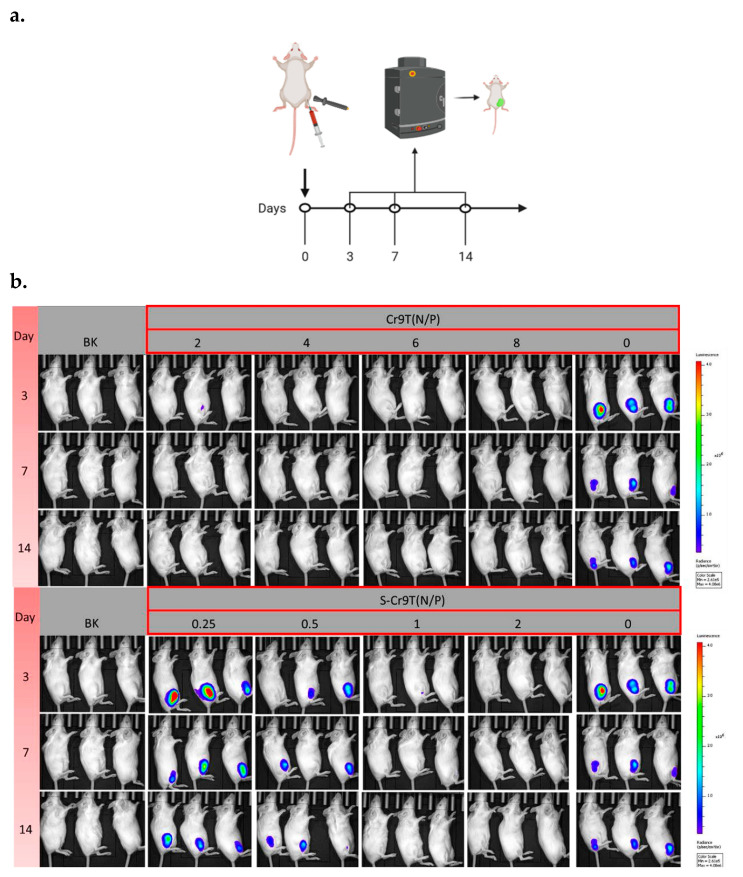
Enhanced intracellular expression of the luciferase gene by S-Cr9T–plasmid complexes. (**a**) Mice were injected with a mixture of CPPs and pNL4.3-luc at varying concentrations. In situ electroporation was applied post-injection. On days 3, 7, and 14 post-immunization, bioluminescence imaging was conducted by administering luciferin, anesthetizing the mice, and recording fluorescence intensity at the injection sites. (**b**) In vivo bioluminescence imaging demonstrates the increase in bioluminescence intensity over days post-immunization with Cr9T and S-Cr9T, indicating the enhanced expression of the luciferase reporter gene. (BK: blank control).

Following the promising in vitro results, we selected S-Cr9T at N/P ratios of 0.25, 0.5, 1, and 2, and Cr9T at N/P ratios of six and eight, for comparative in vivo studies. In these experiments, the luciferase-expressing plasmid pNL4.3-luc was complexed with each peptide and injected into mouse muscle tissue, followed by electroporation to facilitate plasmid uptake (Figure 5a). The intracellular expression of luciferase was monitored at the injection site on days 3, 7, and 14 post-injections. Notably, the S-Cr9T complex at an N/P ratio of 0.25 exhibited the highest levels of luciferase expression, approximately doubling the expression observed with the naked plasmid (Figure 5b and Figure S3a–d). This marked improvement in intracellular expression highlights the efficacy of S-Cr9T in enhancing plasmid delivery and gene expression. In contrast, the Cr9T complex did not demonstrate a similar enhancement in intracellular expression, indicating that while Cr9T facilitates plasmid entry, it may not be as effective as S-Cr9T in driving high levels of intracellular antigen production. These results suggest that S-Cr9T encapsulation significantly boosts the intracellular expression of delivered plasmids, particularly at an optimal N/P ratio of 0.25, making it a more effective carrier for in vivo gene delivery compared to Cr9T.

### 3.6. S-Cr9T-pOVA Plasmid Complex Induces Robust Splenocyte IFN-γ Secretion and Antibody Responses Following Immunization

**Figure 6:**
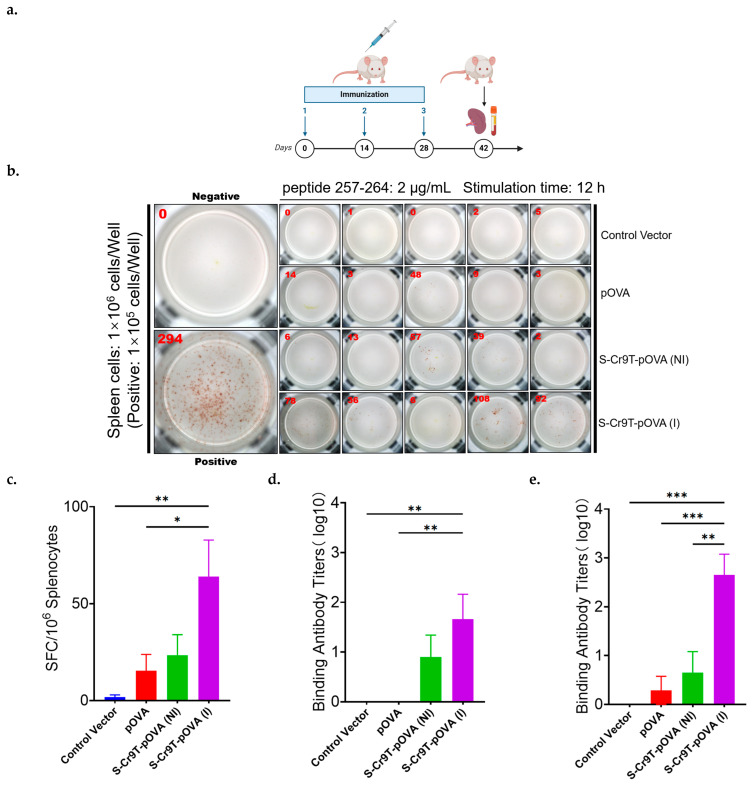
S-Cr9T-pOVA plasmid complex induces robust lymphocyte IFN-γ secretion and enhanced antibody responses following immunization. (**a**) Immunization and sample collection schedule: 25 µg of pVAX-OVA was injected intramuscularly (I.M.) into each mouse (n = 8) with a two-week interval between each of the three immunizations. The groups included a blank vector group, naked plasmid group, non-incubated S-Cr9T+pOVA group (NI), and incubated S-Cr9T+pOVA group (I). (**b**,**c**) ELISPOT assays for IFN-γ expression in splenocytes demonstrated significant differences between the naked plasmid group and the incubated S-Cr9T+pOVA group (* *p* < 0.05, ** *p* < 0.01, unpaired test). (**d**,**e**) OVA-specific IgG binding antibodies after the second immunization (** *p* < 0.01, unpaired test) and after the third immunization (** *p* < 0.01, *** *p* < 0.001, unpaired test).

To evaluate the immunogenicity of the S-Cr9T-pOVA complex, we immunized mice via intramuscular injection with this complex and assessed antibody production and cellular immune responses. Antibody levels were measured after the second and third immunizations, and IFN-γ secretion by spleen cells was evaluated following the third immunization (Figure 6a). Based on the in vitro results, an N/P ratio of 0.25 was selected for the in vivo experiments due to its pronounced effects. To ensure stable complex formation, S-Cr9T and the plasmid were preincubated prior to injection in one group (denoted as ‘I’), allowing sufficient time for interaction and the formation of a well-defined complex. In comparison to both the naked plasmid and the mixture injected immediately after combining S-Cr9T and plasmid without preincubation (denoted as ‘NI’), the preincubated complex (I) elicited significantly stronger immune responses. Specifically, IFN-γ secretion by splenocytes in the S-Cr9T-pOVA group was more than threefold higher than in the naked plasmid group, indicating a substantially stronger lymphocyte response (Figure 6b,c). In addition to the robust cellular response, antibody levels induced by the S-Cr9T-pOVA complex were significantly elevated. After the second immunization, antibody titers were over 80-fold higher compared to the naked plasmid group, and after three immunizations, they were more than 500-fold higher (Figure 6d,e). These findings highlight the critical role of complex formation in enhancing both humoral and cellular immune responses, as the non-incubated (NI) group showed a markedly weaker immune response. While these results demonstrate the considerable potential of the S-Cr9T complex to improve plasmid delivery and immune activation, further optimization is needed to maximize immune efficacy. Nevertheless, the data suggest promising prospects for the future development of this system in vaccine applications.

## 4. Discussion

In this study, novel cell-penetrating peptides (CPPs) were successfully designed and engineered by incorporating cysteine linkages and stearyl modifications at the C-terminus. Three candidate CPPs were obtained: Cr9T, S-Btat, and S-Cr9T. These CPPs demonstrated the ability to form stable peptide–plasmid complexes, with the stearylated peptides (S-Btat and S-Cr9T) forming highly stable and uniform structures at lower N/P ratios. Distinct differences in cellular interaction kinetics and transfection efficiency were revealed between Cr9T and its stearylated counterpart S-Cr9T. Cr9T facilitated rapid plasmid internalization at higher N/P ratios (6–10), whereas S-Cr9T exhibited prolonged membrane anchoring and stable complex formation at lower N/P ratios (0.25–2). This prolonged cell adherence contributed to improved transfection efficiency, particularly in serum-containing conditions where serum components paradoxically enhanced plasmid release and expression.

Importantly, although additional toxicity assays were not conducted in this study, the literature indicates that TAT-derived CPPs generally exhibit low cytotoxicity at the concentrations used for intracellular delivery. Previous studies demonstrated minimal chronic toxicity in cultured cells, and reviews highlight the biocompatibility of CPPs [33,34,35]. These findings support the notion that the CPP variant employed in our study is unlikely to introduce significant cellular toxicity.

These findings have important theoretical and practical implications. Mechanistically, the contrasting kinetics between Cr9T and S-Cr9T highlight the role of stearyl modifications in balancing cell adherence and intracellular plasmid release. Prolonged membrane anchoring may favor controlled plasmid release, ultimately enhancing delivery efficacy. Further investigation into endocytic pathways, intracellular trafficking, and the balance between direct membrane penetration and endosomal escape will provide deeper mechanistic insights into CPP-mediated delivery.

From an applied perspective, our results demonstrate the potential of CPPs, particularly S-Cr9T, as efficient carriers for DNA vaccines. Both in vitro and in vivo experiments showed significant transfection efficiency, with S-Cr9T achieving robust gene expression without reliance on electroporation. The stable distribution and prolonged expression activity observed further validate its applicability for DNA vaccine delivery.

However, some limitations remain. While S-Cr9T enhanced gene expression efficiency, its ability to improve humoral immune responses remains limited. This constraint may reduce its suitability for vaccine platforms that require strong antibody responses. In addition, the stability of CPP–plasmid complexes under physiological conditions and the durability of immune responses warrant further investigation.

## 5. Conclusions

This study demonstrates that stearyl-modified CPPs, particularly S-Cr9T, significantly enhance the delivery and immunogenicity of DNA vaccines. The formation of stable complexes with DNA by S-Cr9T resulted in improved cellular uptake and plasmid expression. These findings highlight the potential of S-Cr9T as an effective adjuvant in DNA vaccine development, offering a promising solution to the longstanding challenges of cellular uptake and immunogenicity. This advancement represents a significant step in the design and application of CPPs for future vaccine development and gene therapy.

## Figures and Tables

**Table 1:** Structural peaks of peptides forming polymers before and after gelation.

Before Gel Formation			
		Mean ± SD	% Intensity
Btat	Peak 1	0.6213 ± 0.2880	20.3
	Peak 2	3.615 ± 3.137	34.9
	Peak 3	141.8 ± 447.0	44.8
S-Btat	Peak 1	0.6213 ± 0.03785	0.2
	Peak 2	1.294 ± 0.3040	11.5
	Peak 3	15.69 ± 5.891	37.8
R9Tat	Peak 1	1.499 ± 0.9809	61.1
	Peak 2	37.84 ± 34.6	20.9
	Peak 3	2669 ± 1400	18
Cr9T	Peak 1	3.122 ± 2.639	60.9
	Peak 2	32.67 ± 47.07	27.4
	Peak 3	1281 ± 1309	11.7
S-Cr9T	Peak 1	43.82 ± 8.854	26.3
	Peak 2	255 ± 51.46	73.7
	Peak 3	0 ± 0	0
**After Gel Formation** **(12 h)**			
		Mean ± SD	% Intensity
Btat	Peak 1	4.849 ± 6.949	48.4
	Peak 2	105.7 ± 11.2	24.8
	Peak 3	2669 ± 1430	26.8
S-Btat	Peak 1	3.122 ± 0.6848	3.4
	Peak 2	15.69 ± 6.931	39.1
	Peak 3	164.2 ± 118.7	49.1
R9Tat	Peak 1	0.7195 ± 0.3133	31.3
	Peak 2	58.77 ± 43.34	31.8
	Peak 3	5560 ± 1004	36.9
Cr9T	Peak 1	5.615 ± 5.104	46.3
	Peak 2	255 ± 314.1	53.7
	Peak 3	0 ± 0	0
S-Cr9T	Peak 1	18.17 ± 8.625	62.8
	Peak 2	255 ± 118.7	33.4
	Peak 3	5560 ± 770.7	3.8

Notes: Mean ± SD: The particle size of the peak (nm)%; each measurement was repeated three times. Intensity: The ratio of different peaks in the mixture.

**Table 2:** The particle size of peptides before and after gelation.

Before Gel Formation		
	Repeats	Z Avg d.nm
Btat	1	10.02
	2	10.86
	3	20.91
S-Btat	1	21.48
	2	81.03
	3	22.1
R9Tat	1	7.146
	2	309.5
	3	6.423
Cr9T	1	6.857
	2	6.739
	3	7.052
S-Cr9T	1	667.7
	2	438
	3	491
**After Gel Formation (12 h)**		
	Repeats	Z Avg d.nm
Btat	1	22.71
	2	63.17
	3	20.97
S-Btat	1	30.8
	2	31.13
	3	35.07
R9Tat	1	186.7
	2	164.8
	3	163.8
Cr9T	1	16.59
	2	98.26
	3	18.92
S-Cr9T	1	32.82
	2	65.68
	3	51.25

Notes: Z: Zeta potential. Avg d.nm: Average particle size (nm).

**Table 3:** Structural peaks of peptide–plasmid complexes.

CPP–Plasmid Complex	N/P		Mean ± SD	% Intensity
S-Cr9T	0.5	Peak 1	15.69 ± 4.647	2.4
		Peak 2	190.1 ± 190.7	97.6
		Peak 3	0 ± 0	0
	1	Peak 1	164.2 ± 106.7	100
		Peak 2	0 ± 0	0
		Peak 3	0 ± 0	0
	2	Peak 1	7.531 ± 1.924	7.4
		Peak 2	255 ± 190	92.6
		Peak 3	0 ± 0	0
Btat	0.5	Peak 1	396.1 ± 206.6	100
		Peak 2	0 ± 0	0
		Peak 3	0 ± 0	0
	1	Peak 1	342 ± 113.2	100
		Peak 2	0 ± 0	0
		Peak 3	0 ± 0	0
	2	Peak 1	10.10 ± 5.835	19.8
		Peak 2	190.1 ± 222.6	50.2
		Peak 3	3580 ± 1379	30
S-Btat	0.5	Peak 1	393.76 ± 86.23	70.40
		Peak 2	6.8 ± 1.39	29.60
		Peak 3	0 ± 0	0.00
	1	Peak 1	716.77 ± 186.39	75.43
		Peak 2	8.4 ± 0.52	24.57
		Peak 3	0 ± 0	0.00
	2	Peak 1	18.57 ± 1.13	77.13
		Peak 2	550.13 ± 63.85	21.90
		Peak 3	0.79 ± 1.12	1.00
R9Tat	0.5	Peak 1	249.77 ± 76.37	91.73
		Peak 2	1379.33 ± 261.7	8.27
		Peak 3	0 ± 0	0.00
	1	Peak 1	219.63 ± 88.92	90.43
		Peak 2	1609.62 ± 2271.5	9.57
		Peak 3	0 ± 0	0.00
	2	Peak 1	178.2 ± 37.43	83.97
		Peak 2	4290.33 ± 154.01	16.03
		Peak 3	0 ± 0	0.00
Cr9T	0.5	Peak 1	74.21 ± 10.61	100.00
		Peak 2	0 ± 0	0.00
		Peak 3	0 ± 0	0.00
	1	Peak 1	270.66 ± 53.29	100.00
		Peak 2	0 ± 0	0.00
		Peak 3	0 ± 0	0.00
	2	Peak 1	123.63 ± 16.33	100.00
		Peak 2	0 ± 0	0.00
		Peak 3	0 ± 0	0.00

Notes: Mean ± SD: The particle size of the peak (nm) %; each measurement was repeated three times. Intensity: The ratio of different peaks in the mixture.

**Table 4:** Particle sizes of CCP–plasmid complexes.

CCP–Plasmid Complex	N/P	Repeats	Z Avg d.nm
S-Cr9T	0.5	1	143.6
		2	138.9
		3	137.3
	1	1	131.2
		2	176.3
		3	167.9
	2	1	197.1
		2	255.5
		3	260.8
S-Btat	0.5	1	376.9
		2	380
		3	379.1
	1	1	283.2
		2	234.5
		3	217.5
	2	1	113.4
		2	200.8
		3	93.41
Btat	0.5	1	714.2
		2	773.2
		3	856.2
	1	1	594.3
		2	730.8
		3	585.5
	2	1	319.0
		2	417.2
		3	416.9
R9Tat	0.5	1	165.7
		2	156.0
		3	156.3
	1	1	161.2
		2	265.6
		3	151.1
	2	1	224.3
		2	196.9
		3	165.2
Cr9T	0.5	1	1730.0
		2	1893.0
		3	940.2
	1	1	479.9
		2	733.0
		3	612.6
	2	1	537.7
		2	441.7
		3	623.8
Plasmid Only	0	1	181.6
		2	156.1
		3	153.7

Notes: Z: Zeta potential. Avg d.nm: Average particle size (nm).

## Data Availability

All data reported in this paper will be shared by contacting the lead contact upon request. Any additional information required to reanalyze the data reported in this work is available from the lead contact upon request.

## Supplementary Materials

The following supporting information can be downloaded at https://www.mdpi.com/article/10.3390/vaccines13010094/s1: Figure S1. Test of Gelation; Figure S2. In vivo Bioluminescence Imaging of Muscle Gene Expression; Figure S3. Cellular Uptake of CPP–Plasmid Complexes over Time.
