# Supplementary material for: Enhancing DNA Vaccine Delivery Through Stearyl-Modified Cell-Penetrating Peptides: Improved Antigen Expression and Immune Response In Vitro and In Vivo
Source: Vaccines (Basel). 2025 Jan 20;13(1):94. doi: 10.3390/vaccines13010094 (PMC11768954; doi:10.3390/vaccines13010094)
Supplement: Supplementary file 1 [file vaccines-13-00094-s001.zip › vaccines-3352712-supplementary.pdf]

SUPPLEMENTARY FIGURES

Figure S1. Test of Gelation.

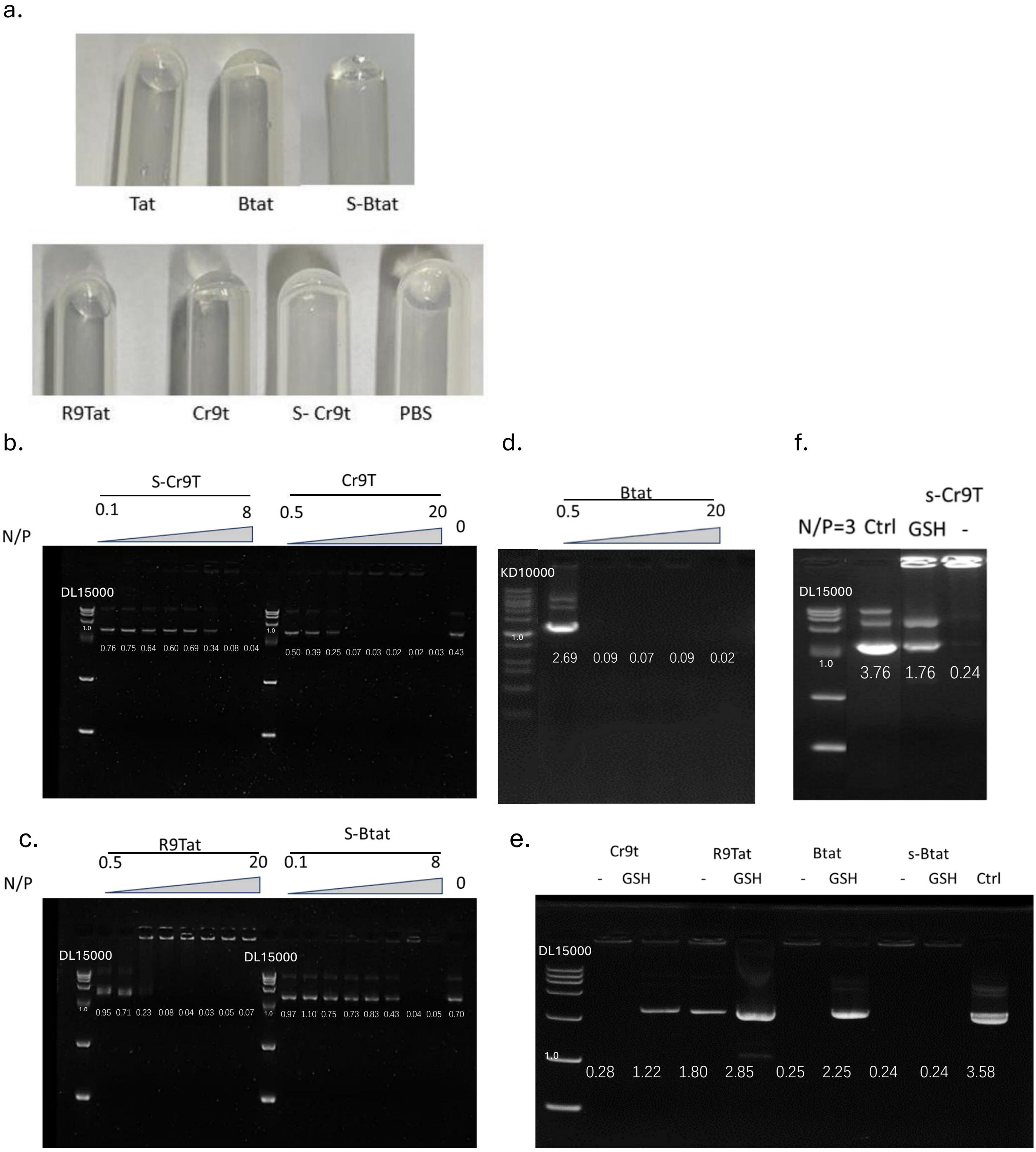

Panel a: Peptides were first dissolved in DMSO at 100 mM, then diluted to 30 mM with 1x PBS. The solutions were incubated on a rotating shaker at 240 rpm at room temperature to induce gelation. PBS, R9Tat, and Tat presented as liquids, while all other substances formed gels (non-gel samples flowed to the sidewall, while gels remained at the bottom.) **Panels b-d:** DNA agarose gel electrophoresis of cell-penetrating peptide-plasmid complexes. The gels were formed in vitro, followed by incubation with diluted peptide solutions to create the complexes. Complete encapsulation of plasmids was observed at N/P ratios exceeding 2. The numbers on the gel image indicate the relative optical density values compared to the marker. **Panels e-f:** After mixing peptide-plasmid complexes (N/P = 3) with 10 mM reduced GSH solution, the mixture was incubated for 30 min at room temperature. This treatment cleaved disulfide bonds in the CCPs, leading to plasmid release. The process was visualized using DNA agarose gel electrophoresis. The numbers on the gel image indicate the relative optical density values compared to the marker.

Figure S2: Cellular Uptake of CPP-Plasmid Complexes Over Time.

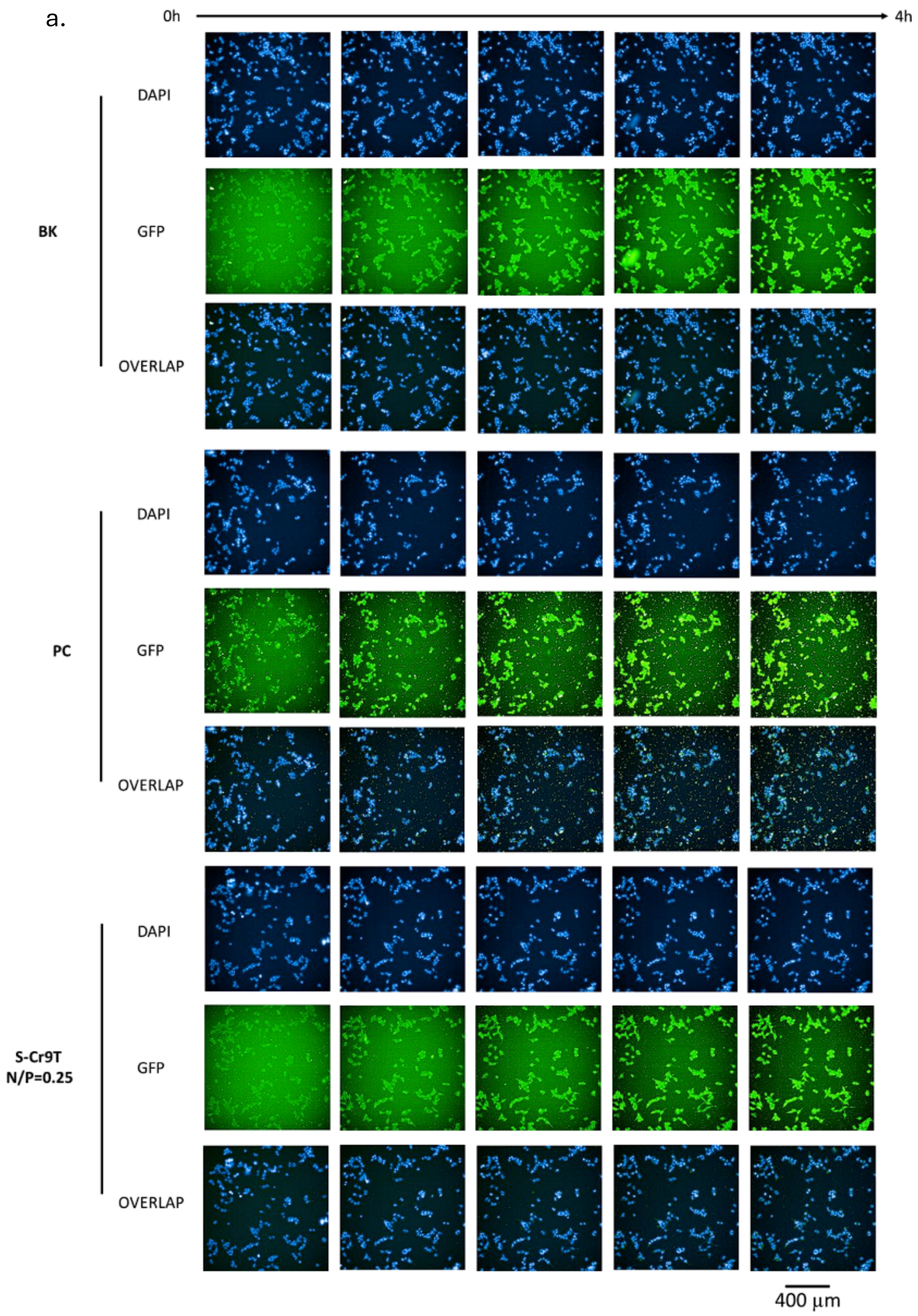
a.
0h
4h
BK
DAPI
GFP
OVERLAP
PC
DAPI
GFP
OVERLAP
S-Cr9T
N/P=0.25
DAPI
GFP
OVERLAP
400 µm

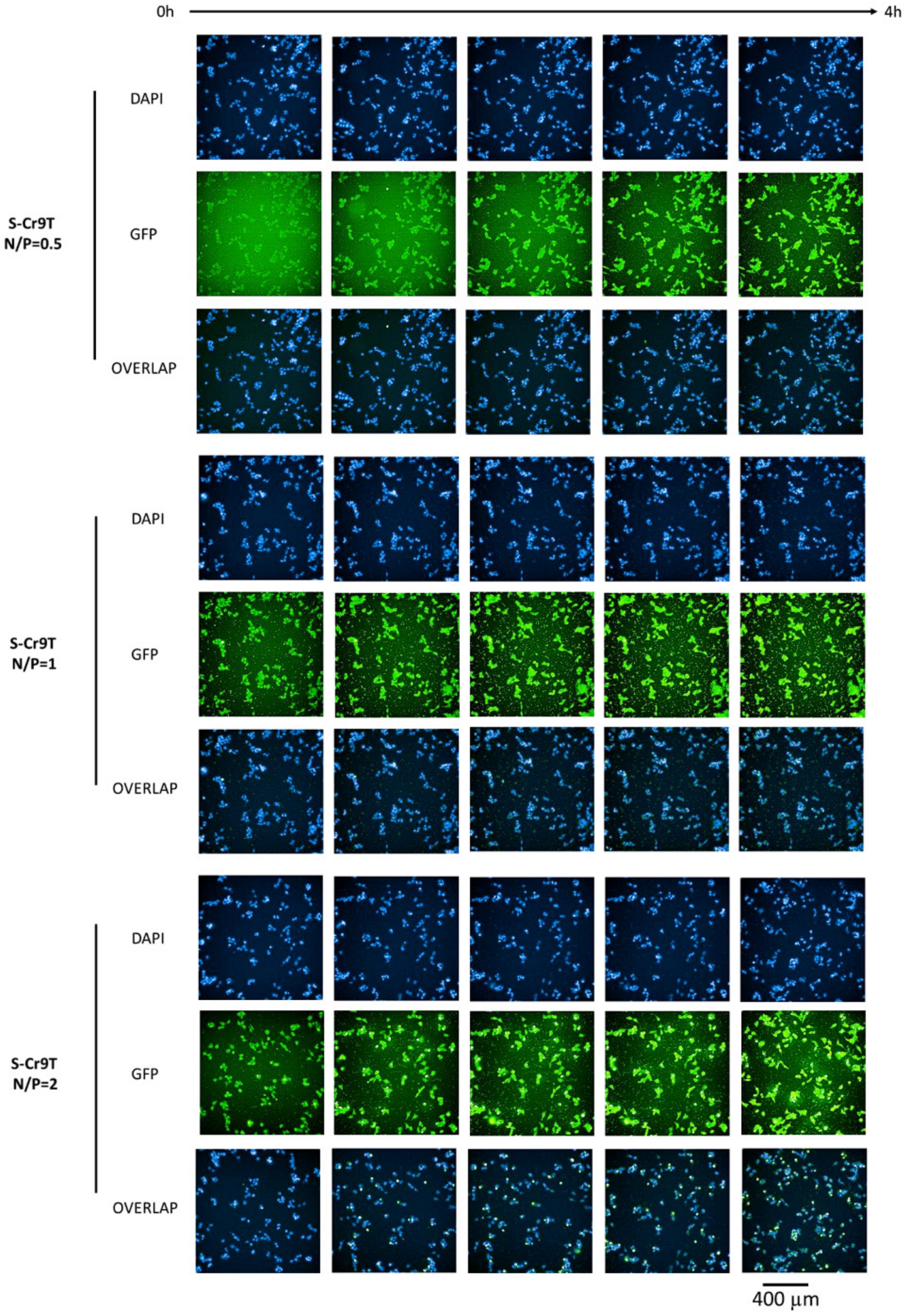
0h
4h
S-Cr9T
N/P=0.5
DAPI
GFP
OVERLAP
S-Cr9T
N/P=1
DAPI
GFP
OVERLAP
S-Cr9T
N/P=2
DAPI
GFP
OVERLAP
400 μm

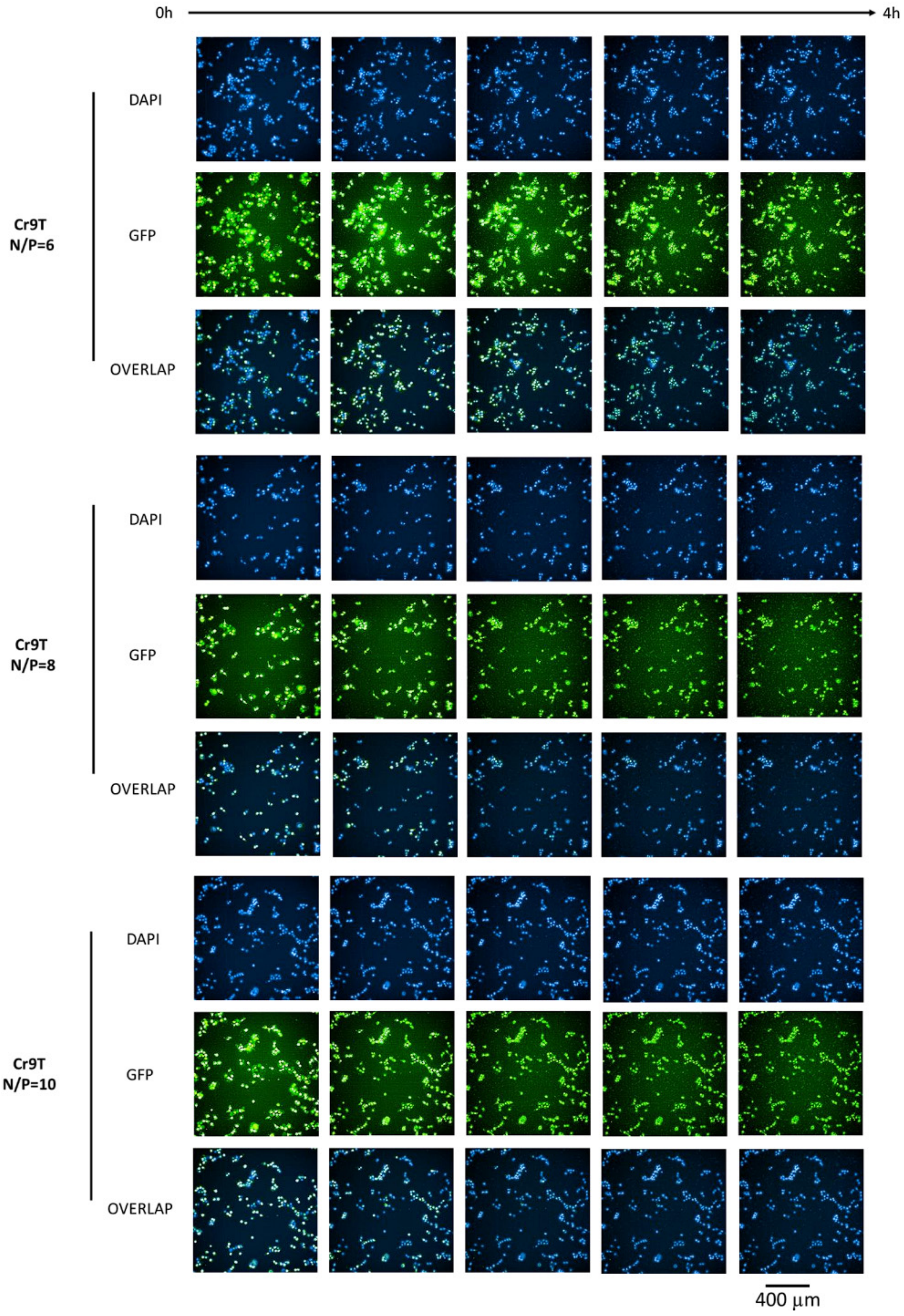
0h
4h
Cr9T
N/P=6
DAPI
GFP
OVERLAP
Cr9T
N/P=8
DAPI
GFP
OVERLAP
Cr9T
N/P=10
DAPI
GFP
OVERLAP
400 μm

b.

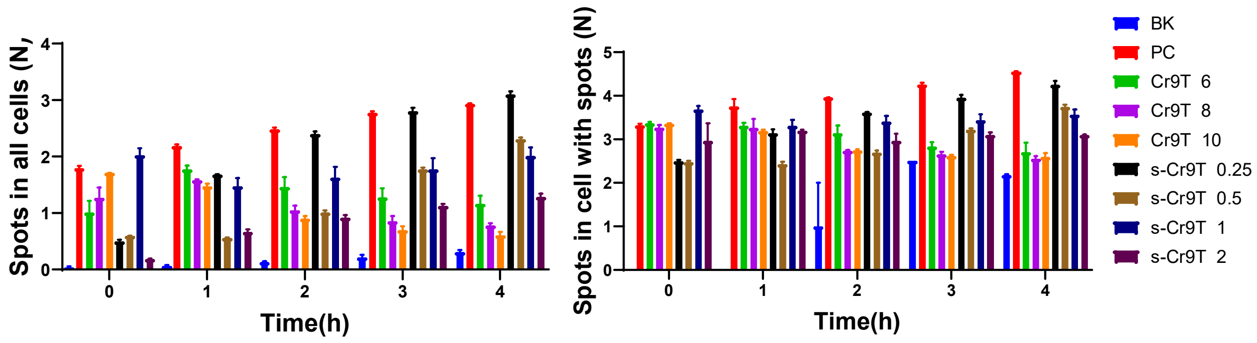

a. The plasmids were pre-stained with picogreen before reacted with CPPs and intended to reveal plasmid-CPP complex directly attachment with cultured HEK293T cells. The stained plasmids were then complexed with CPPs at various N/P ratios (ratio of positive charges on CPPs to negative charges on plasmids). Fluorescence images were acquired under physiological conditions (37°C and 5% CO_2) to mimic the cellular environment. Cells cultured at 37°C and 5% CO_2 were used to react with CPP-plasmid-picogreen labeled complexes (Cr9T or S-Cr9T) over 4 h. Images were captured at 0, 1, 2, 3, and 4 h after mixing the CPP-plasmid complexes with cells. Cellular localization of the complexes was tracked to assess cellular uptake efficiency. Cell nuclei were stained with DAPI, appearing blue in the images. Cell membranes exhibited some degree of autofluorescence. Plasmid DNA, identified as bright green fluorescent spots, was localized and counted to quantify cellular uptake at each time point. BK, Blank (no CPP or plasmid); PC, Plasmid with the transfection reagent, TransIT®-LT1. **b.** High-content fluorescence results for CR9t and S-Cr9T, including spots in all cells and spots in cells with spots. (BK: blank control, PC: positive control).

Figure S3. In vivo Bioluminescence Imaging of Muscle Gene Expression.

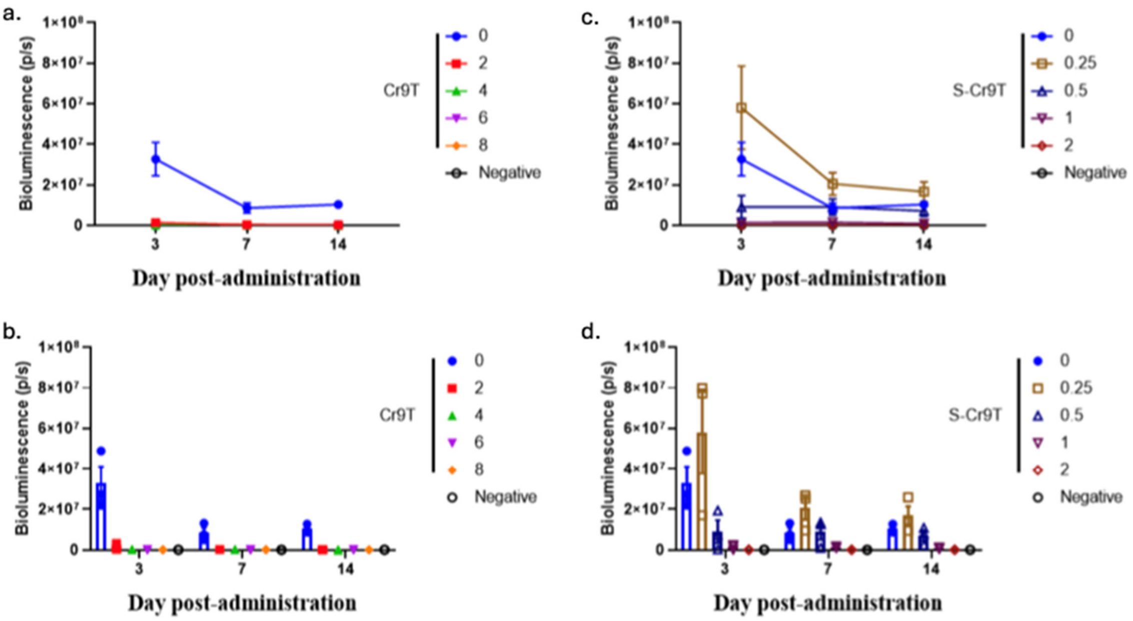

Plasmid pNL4.3-luc, encoding the luciferase reporter gene, was complexed with the selected cell-penetrating peptides (Cr9T and S-Cr9T) to facilitate delivery into the muscle cells of mice. The complexes were then injected intramuscularly. Bioluminescence imaging was used to monitor luciferase activity, which serves as a reporter for plasmid expression within the muscle tissue. **a, b:** Line and bar graphs depict the increase in bioluminescence intensity over days post-immunization for the Cr9T peptide. **c, d:** Line and bar graphs depict the increase in bioluminescence intensity over days post-immunization for the S-Cr9T peptide.
